# CT-based multi-regional radiomics model for predicting contrast medium extravasation in patients with tumors: A case-control study

**DOI:** 10.1371/journal.pone.0314601

**Published:** 2025-03-10

**Authors:** Lili Hu, Jingjing Zhang, Xiaofei Wu, Wenbo Xu, Zi Wang, Heng Zhang, Shudong Hu, Yuxi Ge

**Affiliations:** 1 Department of Radiology, Affiliated Hospital of Jiangnan University, Wuxi, Jiangsu, China; 2 Department of Emergency, Jiangnan University Affiliated Central Hospital, Wuxi, Jiangsu, China; 3 Wuxi Research Institute, Fudan University, Wuxi, Jiangsu, China; 4 Institute of Translational Medicine, Wuxi, Jiangsu, China; University of Pisa, ITALY

## Abstract

**Objective:**

To develop a non-contrast CT based multi-regional radiomics model for predicting contrast medium (CM) extravasation in patients with tumors.

**Methods:**

A retrospective analysis of non-contrast CT scans from 282 tumor patients across two medical centers led to the development of a radiomics model, using 157 patients for training, 68 for validation, and 57 from an external center as an independent test cohort. The different volumes of interest from right common carotid artery/right internal jugular vein, right subclavian artery/vein and thoracic aorta were delineated. Radiomics features from the training cohort were used to calculate radiomics scores (Rad scores) and develop radiomics model. Non-contrast CT radiomics features were combined with clinical factors to develop an integrated model. A nomogram was created to visually represent the integration of radiomic signatures and clinical factors. The model’s predictive performance and clinical utility were evaluated using receiver operating characteristic (ROC) curve analysis and decision curve analysis (DCA), respectively. Calibration curves were also used to assess the concordance between the model-predicted probabilities and the observed event probabilities.

**Results:**

Thirteen radiomics features were selected to determine the Rad score. The radiomic model outperformed the clinical model in the training, validation, and external test cohorts, achieving a greater area under the ROC curve (AUC) with values of 0.877, 0.866, 0.828 compared to the clinical model’s 0.852, 0.806, 0.740. The combined model yielded better AUC of 0.945, 0.911, and 0.869 in the respective cohorts. The nomogram identified females, the elderly, individuals with hypertension, long term chemotherapy, radiomic signatures as independent risk factors for CM extravasation in patients with tumors. Calibration and DCA validated the high accuracy and clinical utility of this model.

**Conclusions:**

Radiomics models based on multi-regional non-contrast CT image offered improved prediction of CM extravasation compared with clinical model alone.

## Introduction

Contrast-enhanced computed tomography (CECT) is a widely utilized non-invasive diagnostic tool in clinical settings that plays an essential role in oncology by facilitating tumor diagnosis, TNM staging, treatment evaluation, prognosis prediction [1–4], and, most importantly, monitoring treatment response [5]. However, contrast medium (CM) extravasation is a potential complication during CECT when injecting CM under high pressure, with a reported incidence rate of 0.1–1.2% [6]. CM extravasation can result in serious complications, including compartment syndrome, skin ulcers, and tissue necrosis. Extravasated CM may also damage the surrounding vasculature, which induces local inflammatory responses that pose significant health threats [7]. Consequently, careful measures are necessary to mitigate the risk of CM extravasation during CECT procedures in patients with tumors.

CM leaks from blood vessels due to vascular injury caused by injection pressure. Therefore, maintaining the integrity of venous and arterial vessels is crucial to avoiding CM extravasation. Chemotherapy drugs, including vinblastine, paclitaxel, anthracyclines, and platinum-based agents, may increase the risk of CM extravasation. They cause vascular endothelial dysfunction, reduce vascular elasticity [8,9], and render the vascular walls more fragile during tumor diagnosis and treatment. Consequently, to properly manage the risk of CM extravasation in patients with malignancies, a complete vascular evaluation must be performed prior to a CECT examination. However, the current risk assessment for CM extravasation primarily relies on the subjective experience of nursing staff [10,11]. While this approach is useful for identifying high-risk patients, it is time-consuming and inefficient. Additionally, factors such as communication barriers, varying education levels, and incomplete medical histories complicate pre-injection evaluations for all patients [12]. Furthermore, these methods are unable to assess extravasation risk based on its fundamental cause—the condition of the vasculature, which is critical for accurate risk assessment.

Radiomics can effectively characterize pathophysiological features by extracting high-volume image data beyond visual assessment capabilities [13]. Currently, it is used for tumor diagnosis, differential diagnosis, treatment efficacy evaluation, and survival prediction in oncology [14–17]. Previous studies have utilized radiomics to predict vascular invasion in tumors and to evaluate coronary arteries for assessing the risk of coronary artery disease [18,19]. Existing vascular radiomics research has primarily targeted CECT, with few studies addressing non-contrast CT for vascular evaluation. However, non-contrast CT images contain numerous microscopic vascular heterogeneities, such as plaques, vascular elasticity, inflammation, and other features that are invisible to the naked eye but can be extracted via radiomics [20]. The European Society of Urogenital Radiology (ESUR) guidelines on contrast agents suggest that documenting extravasation using CT scans of the affected region may aid in the management of CM extravasation [21]. This enables a more intuitive and objective assessment of vessel condition, thereby facilitating the prediction of CM extravasation. Therefore, radiomics on non-contrast CT has the potential to help predict CM extravasation in patients with tumors.

In this study, we aimed to establish a prediction model based on multi-regional vascular volumes of interest (VOIs) from non-contrast CT images to assess the risk of CM extravasation in patients with tumors and to evaluate its clinical applicability.

## Methods

This retrospective study (LS2022079) was approved by the Institutional Review Board of the Affiliated Hospital of Jiangnan University and was conducted in accordance with the principles of the Declaration of Helsinki, the date of approval was September 13th, 2023. The requirement for informed consent was waived by the ethics committee, given that all data were anonymised and aggregated, ensuring the privacy and confidentiality of the participants.

### Patients

**Fig 1:**
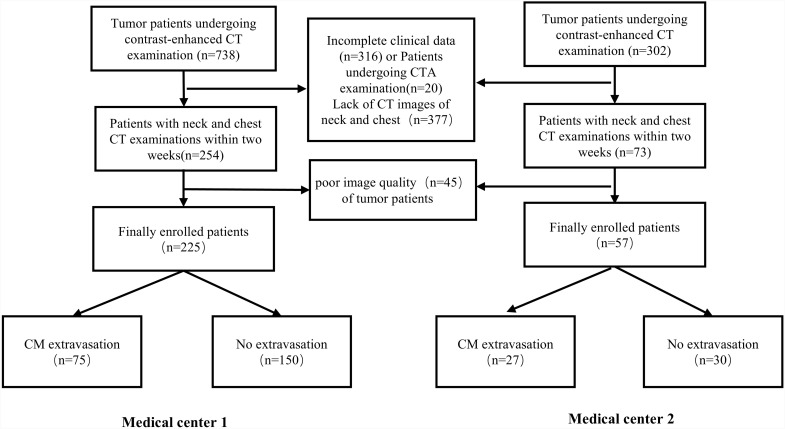
Flow diagram of patient selection.

Between January 1, 2022, and May 31, 2023, a total of 282 tumor patients were enrolled from two medical institutions. Patients were identified using the case management system, the details of the patients with tumors undergo clinical routine CT follow-up examinations are provided in the S1 Fig. At the first institution, 75 patients with tumors with extravasation were matched with 150 patients with tumors non-contrast media (non-CM) extravasation in a 1:2 ratio based on tumor type (S2 Fig). These patients were then divided into a training cohort (n = 157) and a validation cohort (n = 68). Additionally, 57 patients with tumors from the second institution were included as an external test cohort. The data collection and access periods for research purposes began in October 2023.The inclusion criteria were confirmed tumor diagnosis through pathological examination, having undergone CECT examination(s), and comprehensive CT imaging of the neck and chest within 2 weeks before CECT. Patients were excluded if their clinical data were incomplete or if they had undergone CT angiography. The patient selection process sees Fig 1. According to Infiltration Nurses Society Standard [22], the diagnostic criteria of CM extravasation include local reactions, such as ulcer, redness, edema, induration, venous cord-like changes and discomfort.

### Study variables

The primary outcome variable of interest was “CM extravasation”, defined as the accidental leakage of injected fluid into the surrounding tissue. The independent variables in this study included radiomics scores (Rad scores) and clinical characteristics of patients. Rad scores are calculated by extracting the image information of the blood vessel to evaluate the state of blood vessels through quantitative analysis and calculations of vascular images. Clinical characteristics variables included gender, age, BMI, puncture site, types of tumors, chemotherapy cycles, hypertension, diabetes.

### CT data acquisition

CT images were collected using 2 scanners: Somatom Sensation 64 (Siemens Medical Solutions, Forchheim, Germany); or Optima CT660 (GE Medical Systems, Milwaukee, WI, USA). Scanning parameters included the following: slice thickness and spacing, 5 mm; tube voltage, 120 kV; Smart tube current; matrix, 512 ×  512; and pitch, 0.992:1. The scan was performed from the mandible to the diaphragm. According to the injection speed and the patient’s vascular condition, the radiologist nurses choose 22G high-pressure resistant peripheral short catheter (the maximum tolerance pressure is 350 psi). Before intravenous injection of CM, the radiologist nurses performed routine inspection on all venous access again, pre-injected normal saline to determine whether the catheter was unobstructed and confirmed that the catheter was in the vein. Patients should be told to raise their hands to inform the medical staff if there is sudden pain or other discomfort at the injection site during CT high-pressure injection of CM. For enhanced scanning, iohexol was used as the CM, administered at a dose of 0.75 ml/kg using a high-pressure syringe at a rate of 2.5–3.0 ml/s.

### CM extravasation data collection and clinical variable evaluation

The radiology department’s CM extravasation data from January 2022 to May 2023 were searched using the hospital’s adverse event reporting system and the CM extravasation record table. The records of CM extravasation include the Puncture site, injection rate, extravasation volume, local manifestations, patient complaints, intervention measures, and prognosis. The Picture Archiving and Communication System (PACS) retrieved clinical medical records and non-contrast CT images, which were then inspected and analyzed in accordance with inclusion and exclusion criteria. Two radiologists and one nurse participated in the data collection process. The first radiologist (6 years of experience) outlined VOI of vessels on plain CT images with reference to contrast-enhanced CT images. The second radiologist (15 years of experience) was tasked with verifying and rectifying any discrepancies in the delineated regions of interest. Additionally, a nurse was responsible for gathering clinical information of patients in both the CM group and the non-CM group from the medical record system.

### VOI segmentation in different regions

**Fig 2:**
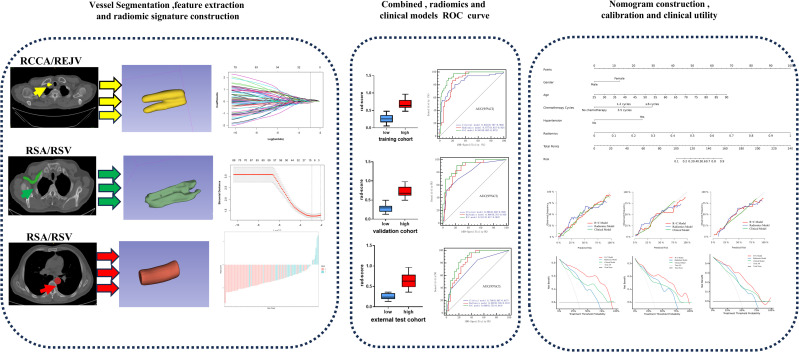
The workflow of a typical radiomics process in study.

The open-source software ITK-SNAP (version 3.4.0, www.itksnap.org) was used for vessel segmentation. A radiologist (ZW) with 7 years’ experience, who was blinded to the clinical data, used ITK-SNAP to outline 5 consecutive layers of the right common carotid artery/right internal jugular vein (RCCA/RIJV), right subclavian artery/vein (RSA/RSV), and thoracic aorta (THA). For each subject, three VOIs were outlined and reviewed by a senior radiologist (SDH, 20 years’ experience). The radiomics process is illustrated in Fig 2.

### Radiomics feature extraction

For the purpose of feature extraction, VOIs were established using a radiomics module that was implemented into the open-source software program 3D Slicer version 4.9 (http://www.slicer.org) and supported by Pyradiomics. Four categories were used to organize the retrieved characteristics: morphological, grayscale statistical, texture, and Gabor wavelet features. These features were employed in the radiomics model to examine the macrovascular state of cancer patients after normalization to a standard range.

### Feature selection and model building

The radiomics model combined features drawn from three groups of large blood vessels. Features possessing nonzero coefficients were chosen to construct the radiomics signature. Multivariate logistic regression was utilized to implement techniques like Maximum Relevance and Minimum Redundancy (mRMR) and Least Absolute Shrinkage and Selection Operator (LASSO) in the primary cohort in order to reduce overfitting and choose the most informative radiomic features for a prediction model. The Rad scores for each patient was determined by fitting the selected features linearly based on their relative coefficients using logistic regression.

### Predictive performance of the radiomics signature

The performance of the radiomics signature, including metrics such as area under the receiver operating characteristic (ROC) curve (AUC), sensitivity, specificity, positive predictive value, negative predictive value, and accuracy, was determined in the training cohort. For consistency, the same optimal cut-off was applied to the validation and external test cohorts.

A new radiomics nomogram model that combines clinical factors and the Rad scores was developed in the training cohort. Then the calibration curves were used to assess the alignment between the predicted risk and the observed outcomes of CM extravasation. Finally, the clinical value of the CT-based nomogram was established using decision curve analysis (DCA).

### Statistical analysis

Statistical evaluations were performed using SPSS version 26.0 (IBM Corp, Armonk, NY, USA) and R version 3.4.2 (R Foundation for Statistical Computing, Vienna, Austria; http://www.Rproject.org). Patient data are expressed as mean values with standard deviation (SD) for continuous variables, and as frequency counts for categorical variables. Chi-squared test (χ²) or Fisher’s exact test was used for comparing categorical variables. Binary logistic regression, odds ratios (OR), and 95% confidence intervals (95% CI) were employed to assess the association between risk factors and the CM extravasation. Based on Rad scores and identified independent predictors of CM extravasation, three models were developed: Model 1 is the clinical model, Model 2 is the radiomics model, and Model 3 is the combined R + C model. Diagnostic capabilities were compared using ROC curve analysis, and AUC was calculated to evaluate model performance. A *p* < 0.05 was considered statistically significant.

## Results

### Characteristics of the patients

282 patients with tumors were included after applying the inclusion and exclusion criteria, there were 180 in the non-CM extravasation group and 102 in the CM extravasation group. The training cohorts comprised 157 patients, the validation cohorts comprised 68 patients, and the external test cohorts comprised 57 patients. There were no significant differences among the three cohorts, except for diabetes (*p* < 0.001) and hypertension (*p* = 0.034), patient characteristics are shown in Table 1.

**Table 1:** Baseline characteristics of patients in three groups.

Variable	Training cohort (n = 157)	Validation cohort (n = 68)	External test cohort (n = 57)	*P* value
**Gender (%)**				0.292
** Male**	81(51.59%)	31(45.59%)	34(59.65%)	
** Female**	76(48.41%)	37(54.41%)	23(40.35%)	
**Age**	63(53,69)	63(56,69)	65(57,69.5)	0.447
**BMI (%)**				0.180
** Normal**	95(60.51%)	33(48.53%)	29(50.88%)	
** Abnormal**	62(39.49%)	35(51.47%)	28(49.12%)	
**Puncture site (%)**				0.944
** Antecubital fossa**	89(56.69%)	40(58.82%)	32(56.14%)	
** Forearm**	68(43.31%)	28(41.18%)	25(43.86%)	
**Types of tumors (%)**				0.636
** Lung cancer**	51(32.48%)	18(26.47%)	18(31.58%)	
** Gastrointestinal tumor**	80(50.96%)	34(50.00%)	31(54.39%)	
** Breast cancer**	26(16.56%)	16(23.53%)	8(14.03%)	
**Chemotherapy cycles (%)**				0.530
**No chemotherapy**	34(21.66%)	19(27.94%)	8(14.03%)	
** 1-2 cycles**	28(17.83%)	14(20.59%)	15(26.32%)	
** 3-5 cycles**	42(26.75%)	16(23.53%)	14(24.56%)	
** ≥6 cycles**	53(33.76%)	19(27.94%)	20(35.09%)	
**Radiotherapy (%)**				0.973
** No**	133(84.70%)	58(85.30%)	49(86.00%)	
** Yes**	24(15.30%)	10(14.70%)	8(14.00%)	
**Hypertension (%)**				0.034 *
** No**	124(78.98%)	50(73.53%)	35(61.40%)	
** Yes**	33(21.02%)	18(26.47%)	22(38.60%)	
**Diabetes (%)**				<0.001*
** No**	143(91.08%)	59(86.76%)	39(68.42%)	
** Yes**	14(8.92%)	9(13.24%)	18(31.58%)	

Note: Data are medians with 25th and 75th percentiles in parentheses for age.

Normal = BMI (Body Mass Index), 18.5-23.9 kg/m^2^, abnormal = BMI < 18.5 kg/m^2^ or > 23.9 kg/m^2, *^ =  p ≤ 0.05.

In the univariable analysis, gender (OR: 2.218, *p* = 0.022), age (OR: 1.07, *p* < 0.001), BMI (OR: 2.16, *p* = 0.026), chemotherapy cycles (OR: 4.133, 7.75, 7.922, *p* = 0.054, *p* = 0.003, *p* = 0.002), hypertension (OR: 11.227, *p* < 0.001), and diabetes (OR: 6.012, *p* = 0.004) were associated with a higher risk of CM extravasation. In addition, multivariable analysis revealed that gender (OR: 2.932, *p* = 0.021), age (OR: 1.076, *p* = 0.002), chemotherapy cycles (OR: 3.380, 7.953, 8.968, *p* = 0.16, *p* = 0.01, *p* = 0.005, *p* = 0.005), and hypertension (OR: 6.356, *p* = 0.001) were independent predictor for CM extravasation (Table 2).

**Table 2:** Univariate and multivariate logistic regression analysis of the clinical risk factors.

Variables	Univariable Analysis		Multivariable Analysis	
	OR	*P* value	OR	*P* value
Gender	2.218(1.124–4.378)	0.022 *	2.932(1.177–7.307)	0.021 *
Age	1.07(1.034–1.108)	< 0.001 *	1.076(1.027–1.128)	0.002 *
BMI	2.16(1.096–4.258)	0.026 *	1.959(0.805–4.768)	0.138
Puncture site	0.742(0.377–1.462)	0.389	–	–
Types of tumors				
Lung cancer	1.00		–	–
Gastrointestinal tumor	1.178(0.557–2.491)	0.668	–	–
Breast cancer	0.972(0.35–2.7)	0.957	–	–
Chemotherapy cycles				
No chemotherapy	1.000		1.000	
1-2 cycles	4.133(0.978–17.464)	0.054	3.380(0.618–18.495)	0.16
3–5 cycles	7.75(2.043–29.402)	0.003 *	7.953(1.639–38.59)	0.01 *
≥6 cycles	7.922(2.151–29.175)	0.002 *	8.968(1.959–41.05)	0.005 *
Radiotherapy	2.325(0.963–5.615)	0.061	–	–
Hypertension	11.227(4.55–27.701)	< 0.001 *	6.356(2.194–18.411)	0.001 *
Diabetes	6.012(1.785–20.243)	0.004 *	1.998(0.419–9.538)	0.385

Note: Data in parentheses are 95% Cis. ^* ^ =  p ≤ 0.05.

### Features selection

**Fig 3:**
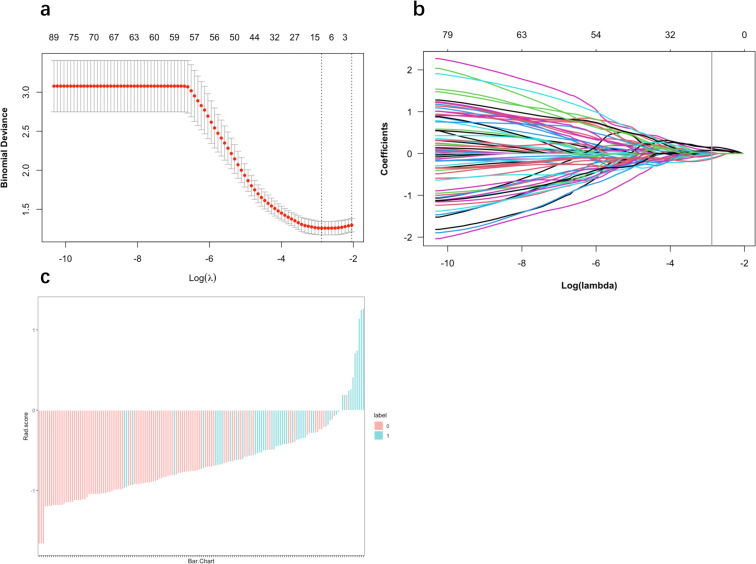
Radiomics features selection with the LASSO logistic regression model and Rad-score distribution bar graph. a The process included selecting tuning parameters (λ) through 10-fold cross-validation, and plotting the binomial deviance against log(λ). The upper x-axis represents the average number of radiomics features, while the lower x-axis shows the log(λ) value. b The LASSO coefficient profiles of the radiomics features were examined. Again, the upper x-axis denotes the average number of radiomics features, and the lower x-axis illustrates the log(λ) value. Dashed lines were drawn at the chosen λ values, and 13 features with non-zero coefficients were selected. c Rad-score distribution bar graph.

Initially, 4278 radiomic features were extracted. After applying sequential feature elimination and conducting a correlation analysis, this number was reduced to a more manageable subset of 667 informative features. Subsequently, the top 13 features were identified using the mRMR and LASSO analyses. These selected features comprised first-order grey histogram metrics, second-order texture descriptors, and higher-order wavelet coefficients; more specifically, 2 were first-order, 1 was texture, and 10 were wavelet-based features. Fig 3 shows the details on feature selection by LASSO.

### Prediction model performance and comparison

**Fig 4:**
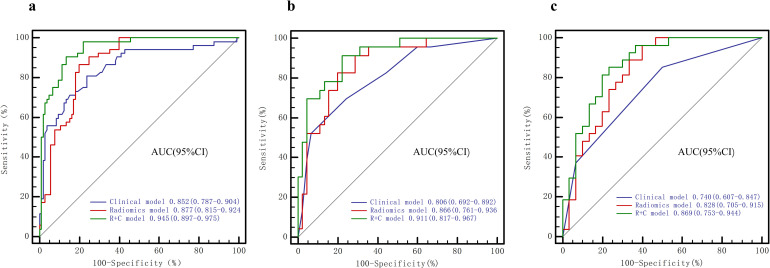
Presents diagnostic performance comparisons by ROC analysis between the (R + C), radiomics, and clinical models in the training, validation, and external test cohorts. Predictive performance of radiomics model in the training, validation, and external test cohorts **(a)**. Predictive performance of the the clinical model, radiomics model, and Rad-score combined with clinical risk factor model (R + C model) was assessed and compared through ROC curves in both the training **(b)**, validation **(c)** and external test cohorts **(d)**.

A radiomic signature was constructed using the 13 distinctive features that were identified in the training cohort. In both the validation cohort and the external test cohort, Radiomics models demonstrated excellent predictive performance, with AUC values of 0.866, 0.828, respectively (Fig 4), with good sensitivity (0.826, 0.963, respectively) and accuracy (0.809, 0.772, respectively). In the validation, and external test cohorts, the R + C model achieved good predictive performance, with AUCs of 0.911, and 0.869, as well as a good sensitivity (0.831, 0.852, respectively) and accuracy (0.824, 0.807, respectively). However, the AUC values of the clinical model were only 0.806 and 0.740 in the validation and external test cohorts, respectively. Detailed evaluation of the performance of these models is presented in Table 3 and Fig 4.

**Table 3:** Performance of the CM extravasation prediction models.

Cohort	Model	AUC (95% CI)	Accuracy	Sensitivity	Specificity	PPV	NPV
	Clinical model	0.852 (0.787–0.904)	0.764	0.808	0.762	0.636	0.886
**Training cohort**	Radiomics model	0.877 (0.815–0.924)	0.809	0.865	0.800	0.692	0.921
	R + C model	0.945 (0.897– 0.975)	0.860	0.904	0.867	0.770	0.940
	Clinical model	0.806(0.692–0.892)	0.794	0.522	0.933	0.800	0.792
**Validation cohort**	Radiomics model	0.866(0.761–0.936)	0.809	0.826	0.800	0.679	0.900
	R + C model	0.911(0.817–0.967)	0.824	0.931	0.778	0.700	0.946
	Clinical model	0.740(0.607–0.847)	0.667	0.852	0.500	0.605	0.789
**External test cohort**	Radiomics model	0.828(0.705–0.915)	0.772	0.963	0.600	0.684	0.947
	R + C model	0.869(0.753–0.944)	0.807	0.852	0.767	0.767	0.852

Note: R + C = combined model (Rad scores combined with clinical risk factor model).

AUC = area under the receiver operating characteristic curve.

NPV= negative predictive value, PPV= positive predictive value.

**Fig 5:**
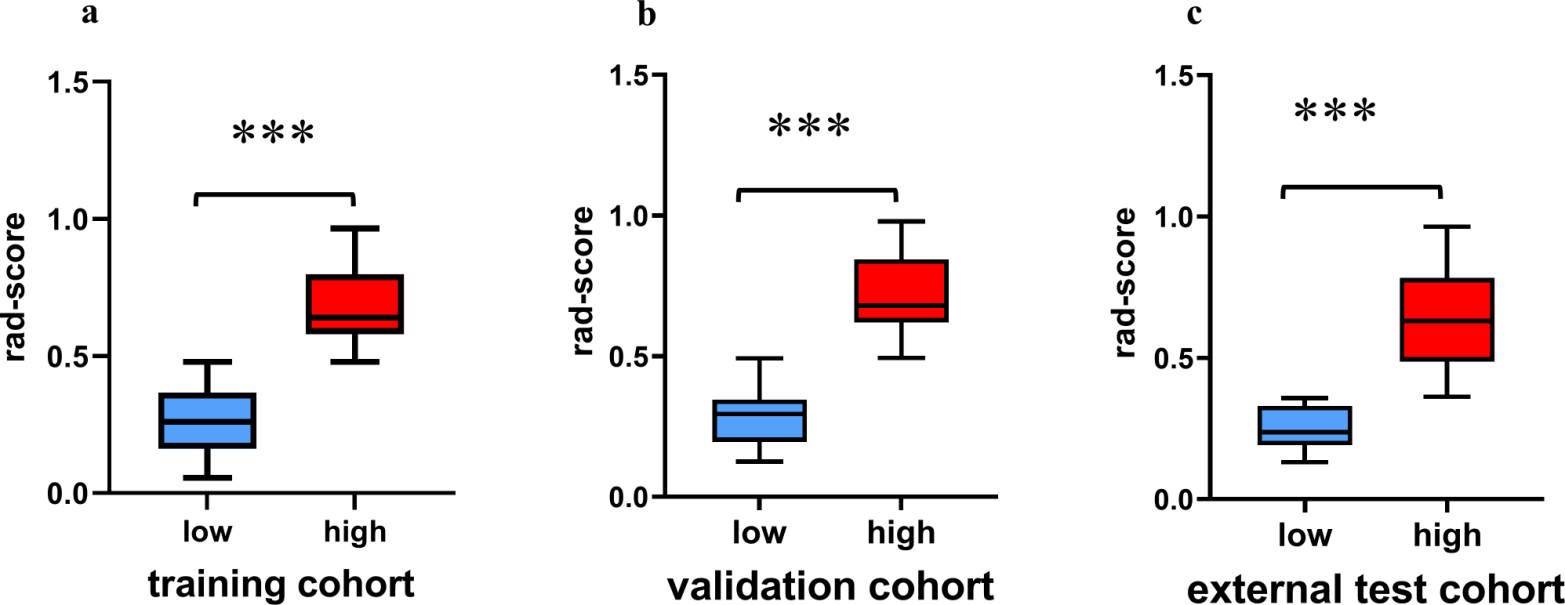
Comparison of radiomic scores between high-risk and low-risk patient groups across the training cohort (a), validation cohort (b), and external test cohort (c). (*=*p <* 0.0001).

Based on the cut-off value derived from the ROC curve, the radiomic signatures were stratified into high- and low-grade groups. Significant variations in Rad-scores were observed between the low- and high-grade patients across the training (*p* <  0.001), validation (*p* <  0.001), and external test (*p* <  0.001) cohorts (Fig 5).

### Calibration and clinical utility

**Fig 6:**
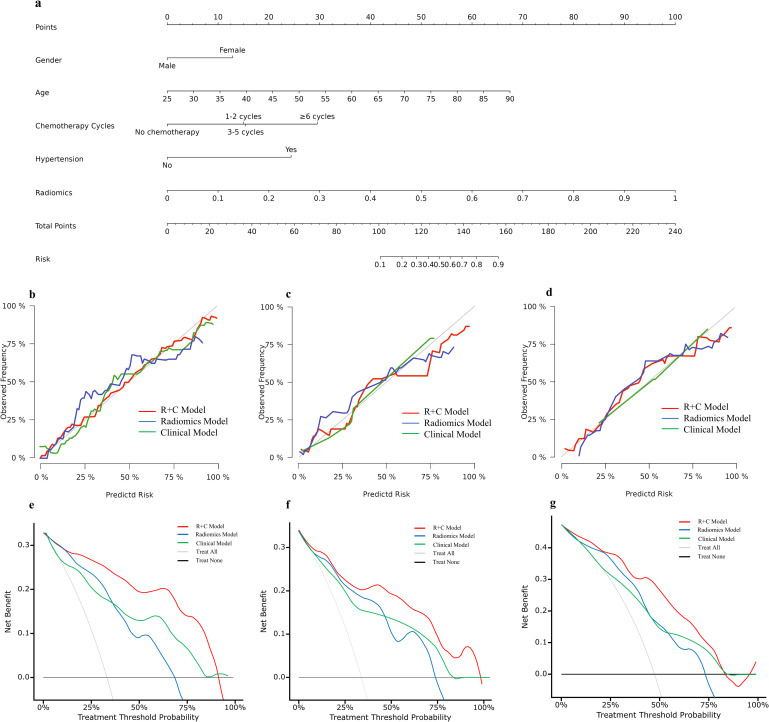
Performance and nomogram of prediction model. A nomogram created using High risk clinical factors of CM extravasation and radiomics score **(a)**, incorporating the gender, age, chemotherapy cycles, and hypertension and radiomics; Calibration curves of clinical model, radiomics model and combined model in the training **(b)**, validation **(c)**, and external test cohorts **(d)**; The DCA of clinical model **(e)**, radiomics model **(f)** and combined model **(g)** in the training, validation, and external test cohorts.

The clinical and radiomics combined model is presented as a nomogram (Fig 6a). Calibration curves were used to evaluate the performance of the proposed model and illustrated the concordance between the probabilities predicted by the model and the actual observed outcomes. In the training cohorts, the calibration curve of the combined model demonstrates a good degree of fit, indicating that the difference between its predicted probabilities and the actual incidence rates is small (Fig 6b-d). In the training, validation, and external test cohorts, the DCA results supported the clinical usefulness of the combined model. The greatest benefits of the combined model model were obtained when the threshold probability was in the range of 25–80%. The use of the nomogram to predict CM extravasation was more effective than was using only clinical variables or Rad-score (Fig 6e-g).

## Discussion

This study aimed to develop a radiomics model to predict CM extravasation in patients with tumors using multi-region vascular delineation from non-contrast CT scans. The model integrates clinical risk factors and radiomics features, demonstrating high predictive efficiency in both internal validation and external testing cohorts. Additionally, a nomogram based on the combined radiomics-clinical model demonstrated significant predictive utility. DCA further highlights the clinical benefits of integrating imaging and clinical variables to predict the risk of CM extravasation.

CECT plays a crucial role in tumor management and is an effective method for assessing treatment efficacy. However, cautious application is required for patients with fragile vasculature and a high risk of CM extravasation. Currently, prevention strategies primarily focus on enhancing assessment and intervention procedures. Traditionally, the injection rate of CM has been a crucial factor in determining the occurrence of CM extravasation. Accurate pre-administration prediction of CM extravasation by radiology technicians could potentially enable a proactive approach to minimize this risk [23]. Specifically, if radiology technicians can accurately determine the probability of CM extravasation prior to CM injection, adjusting the injection rate to a slower pace could significantly reduce the incidence of extravasation events. In this study, we developed a model to predict the risk of CM extravasation by extracting macrovascular features (size, shape, location) and microscopic features (texture, gray-level intensity, fluctuation patterns) from previous non-contrast CT images of patients with tumors. Radiomic analysis outperformed methods based solely on clinical data in predicting the incidence of extravasation within both training and validation cohorts. The combined radiomic-clinical model demonstrated superior predictive accuracy, providing a quantitative method for monitoring CECT extravasation in patients with tumors.

Consistent with previous studies, multivariate analysis confirmed that gender, age, chemotherapy cycles, and hypertension are independent predictors of CM extravasation [7,24,25]. Females have a higher incidence of CM extravasation than males, which may be connected to their estrogen levels. Blood vessel flexibility and permeability may be impacted by estrogen, increasing the likelihood of blood vessel injury during CM injection and raising the risk of extravasation [26]. Age is considered a significant risk factor for CM extravasation. As individuals age, blood vessels undergo several physiological changes, including atrophy, vascular sclerosis, increased susceptibility, and decreased flexibility, which may elevate the risk of CM extravasation in the elderly [27]. Additionally, the response of elderly patients to noxious stimuli is weakened, which may delay the identification and timely treatment of extravasation events [28]. The European Society of Urogenital Radiology (ESUR) guidelines for contrast agents state that frail or damaged blood vessels increase the likelihood of extravasation [21]. In this study, with the increase of chemotherapy cycle, the risk of CM extravasation in tumor patients also increases. This elevation in risk may be due to the vascular damage induced by chemotherapeutic drugs. Repeated exposure to chemotherapeutic agents can cause direct injury to the blood vessels, leading to changes in vascular integrity. With each chemotherapy cycle, the veins may become more susceptible to damage, reducing their capacity to handle the pressure from CM injections, which can lead to an increased risk of extravasation [29]. Hypertension heightens the risk of CM extravasation by inducing vascular changes like decreased elasticity and increased rigidity [30,31]. When large volumes of CM are rapidly injected into vessels that are rigid or occluded, the venous system may exceed its tolerance, potentially leading to vascular rupture and CM extravasation.

This predictive model serves as a valuable tool for nurses to identify patients with tumors who are at a higher risk of CM extravasation. It facilitates appropriate clinical decisions and reduces the likelihood of extravasation events. Objective risk assessment allows for the optimization of care for at-risk patients undergoing CECT [32]. Compared to existing nursing evaluation methods, our model offers a more convenient and efficient estimation of extravasation risk by eliminating the need for intermediate calculations and reducing provider workload. As a result, the model can be used as a daily assessment tool to promptly identify patients with tumors at risk of CM extravasation before undergoing CECT, without increasing staff workload.

While this study provided valuable insights, several limitations need to be acknowledged. First, its retrospective design introduces inherent risks of selection bias, potentially impacting the reliability and representativeness of the results. The retrospective nature of the study leads to potential data being missing or not fully recorded, failing to capture all variables that could affect the outcomes, such as the injection process, patient reactions during examination, materials used for injection, and the technical factors of medical staff, which were not fully evaluated. Second, this study only included patients with lung, gastrointestinal, and breast cancers, which have higher incidence rates, and did not cover all types of tumors that might experience contrast medium extravasation during enhanced CT examinations. This selection may limit the broad applicability of the results. Third, although matching the extravasation group with the non-extravasation group in a 1:2 ratio was used to reduce the impact of data imbalance, while this matching reduced some confounding factors, it could not completely eliminate the potential influence of unconsidered confounding factors on the results. Fourth, the sample size in this study was too small, which may limit the statistical power and generalizability of the findings. Future studies with larger sample sizes and more diverse tumor types are needed to validate and enhance the proposed model.

## Conclusions

In this study, an innovative radiomics methodology based on non-contrast CT was developed, which relied on multi-region vascular delineation, to predict CM extravasation during CECT. The radiomics model exhibited significantly superior predictive efficacy when compared to conventional clinical models. Moreover, the risk for CM extravasation was objectively and quantitatively assessed through the utilization of a nomogram.

## Supporting information

S1 Fig The flowchart of diagnostic process for cervical lymph node metastasis and hepatic metastases. (TIF)

S2 Fig The flowchart of patient data screening and enrollment. (TIF)

S1 Data Data file of training cohort. (XLS)

S2 Data Data file of validation cohort. (XLS)

S3 Data Data file of external test cohort. (XLS)

S4 Data Data file of radiomics. (XLS)

S1 File Description of methods. (PDF)

## Abbreviations

CT: computed tomography
CECT: contrast-enhanced computed tomography
ROC: receiver operating characteristic
AUC: area under the receiver operating characteristic curve
CM: contrast medium
VOI: volumes of interest
DCA: decision curve analysis
LASSO: least absolute shrinkage and selection operator
mRMR: maximum relevance and minimum redundancy
