# Supplementary figures and images for: CT-based multi-regional radiomics model for predicting contrast medium extravasation in patients with tumors: A case-control study
Source: PLoS One. 2025 Mar 10;20(3):e0314601. doi: 10.1371/journal.pone.0314601 (PMC11893132; doi:10.1371/journal.pone.0314601)

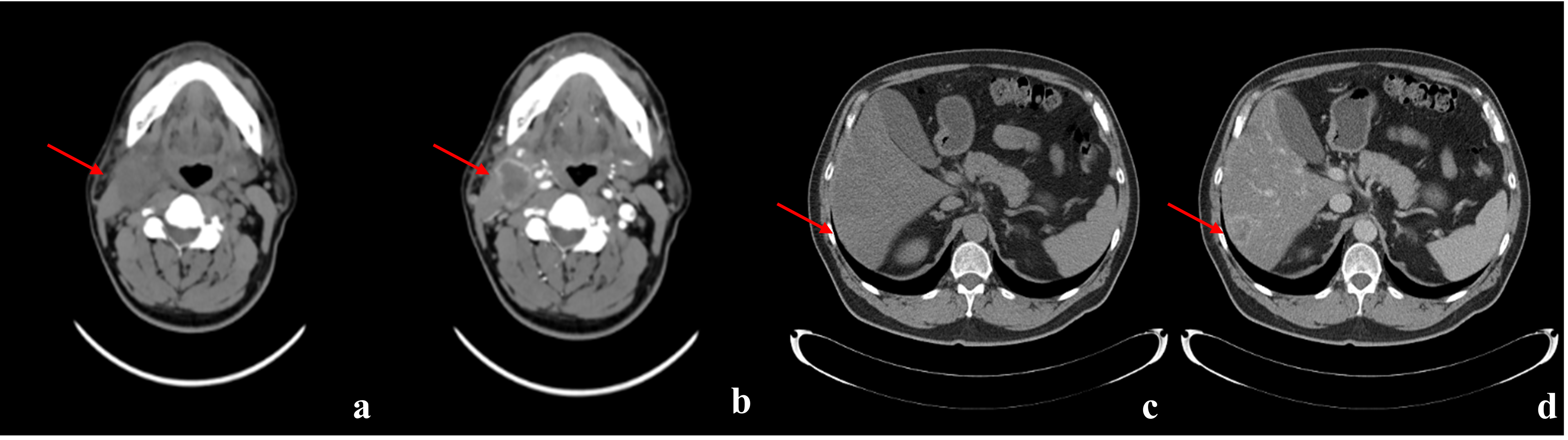

Supplement: S1 Fig — (TIF) [file pone.0314601.s001.tif]

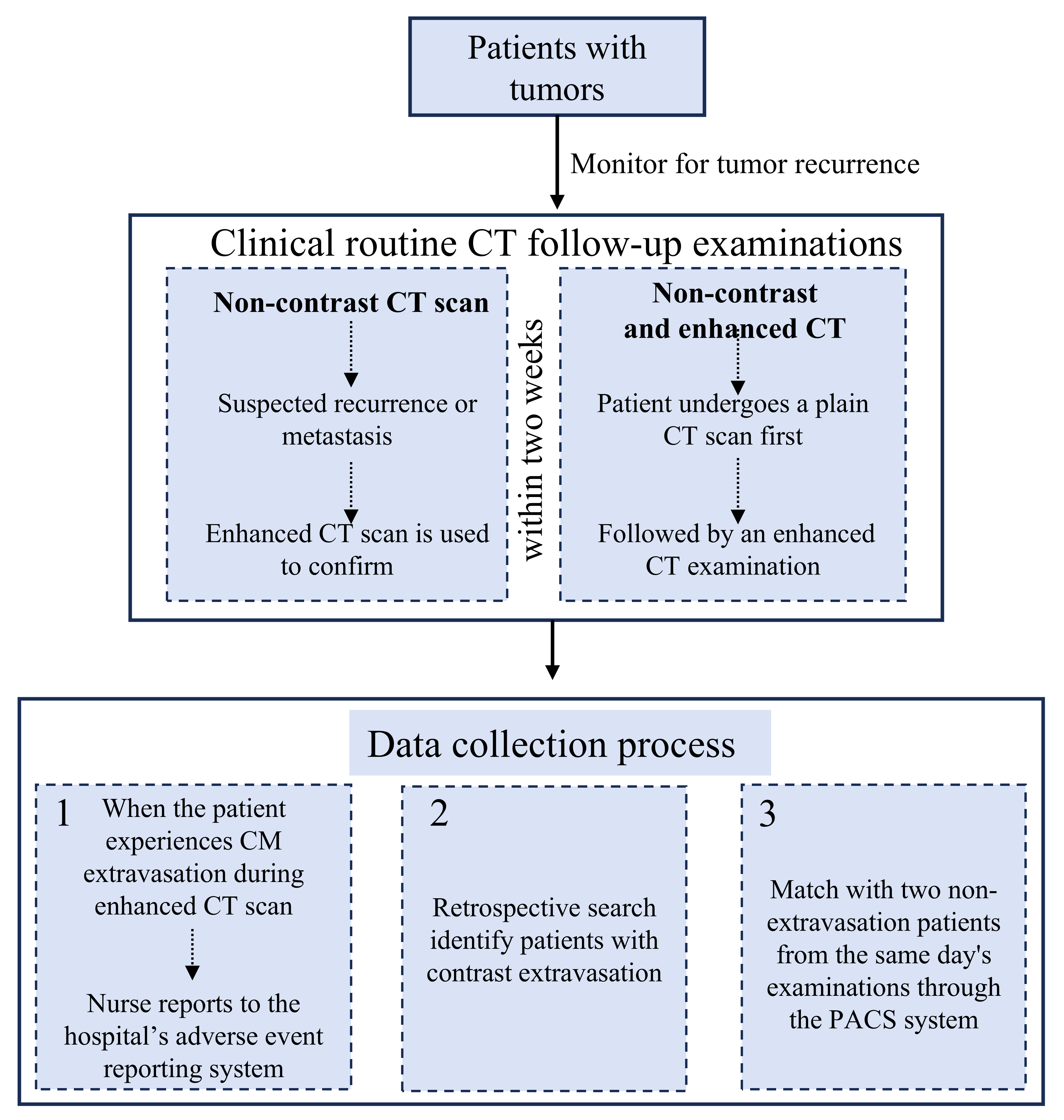

Supplement: S2 Fig — (TIF) [file pone.0314601.s002.tif]
