# Supplementary material for: CT-based multi-regional radiomics model for predicting contrast medium extravasation in patients with tumors: A case-control study
Source: PLoS One. 2025 Mar 10;20(3):e0314601. doi: 10.1371/journal.pone.0314601 (PMC11893132; doi:10.1371/journal.pone.0314601)
Supplement: S1 File — (PDF) [file pone.0314601.s007.pdf]

Supplemental Materials

Methods

Patients

Patients with tumors undergo clinical routine CT follow-up examinations

In this retrospective study, patients were identified using the case management system. The standard procedure involved initial non-contrast CT scans to detect suspicious lesions. Upon identification of such lesions, enhanced CT scans were conducted within a brief interval (usually within two weeks) to determine if the lesions were metastatic. **S1 Fig** demonstrate the diagnostic processes specific to cervical lymph node metastasis and hepatic metastases, respectively. Furthermore, a subset of tumor follow-up patients undergo both non-contrast and enhanced CT scans on the same day during their follow-up examinations. These patients are highly suspected of tumor recurrence, therefore, the oncologist directly request both non-contrast and enhanced CT scans. Consequently, these patients had pre-enhanced non-contrast CT images available. These images were employed to develop predictive models for contrast medium (CM) extravasation through radiomics analysis of the non-contrast CT images.

Patient enrollment process

If CM extravasation occurs during a patient’s enhanced CT scan, the nurse reports it to the hospital’s adverse event reporting system. This system also allows for retrospective retrieval of information regarding patients who experienced leakage events. Therefore, when

identifying a patient with contrast agent extravasation, we match them with two non-extravasation patients of the same tumor type from the same day's examinations via the PACS system to ensure homogeneity among study subjects. **S2 Fig** illustrates the process of the retrospective study design.

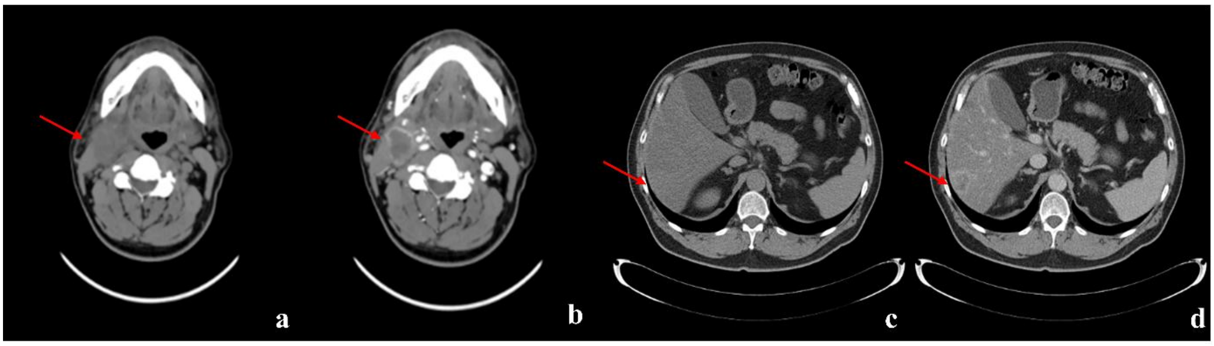

S1 Fig. The flowchart of diagnostic process for cervical lymph node metastasis and hepatic metastases

Initially, the non-contrast CT scan **(a)** did not reveal any significant abnormalities, only showing slight swelling of the soft tissues in the right neck. Five days later, contrast-enhanced CT images **(b)** identified a markedly ring-enhancing enlarged lymph node in the right neck, leading to the diagnosis of a metastatic lymph node. The non-contrast CT **(c)** shows the liver with uniform low density, with no detectable metastatic tumors. The contrast CT **(d)** during the portal venous phase reveals a distinct ring-enhancing metastatic tumor in the right posterior lobe of the liver.

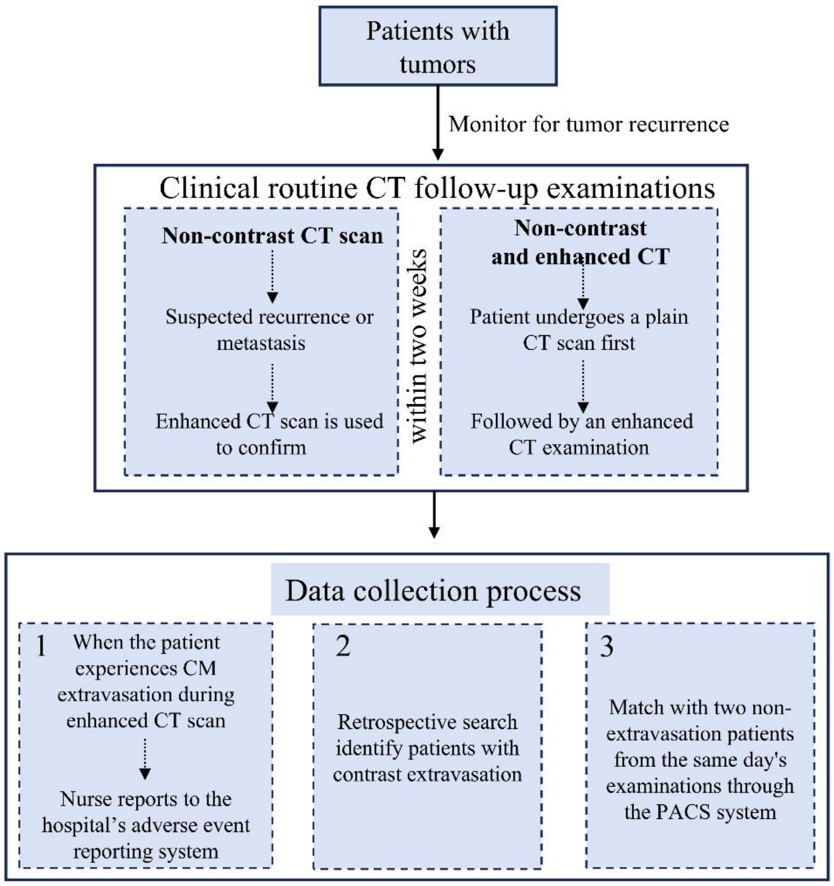

S2 Fig. The flowchart of patient data screening and enrollment
